# Dynamic pathological analysis reveals a protective role against skin fibrosis for TREM2-dependent macrophages

**DOI:** 10.7150/thno.94121

**Published:** 2024-03-11

**Authors:** Yunsheng Liang, Yongfei Hu, Jun Zhang, Haosen Song, Xiaoqian Zhang, Yishan Chen, Yu Peng, Lihua Sun, Yuzhe Sun, Ruzeng Xue, Suyun Ji, Chuanwei Li, Zhili Rong, Bin Yang, Yingping Xu

**Affiliations:** 1 Institute of Dermatology and Venereology, Dermatology Hospital, Southern Medical University, Guangzhou 510091, China.; 2 Department of Dermatology, Dermatology Hospital, Southern Medical University, Guangzhou 510091, China.; 3 Department of Gynecology and Obstetrics, Nanhai Hospital, Southern Medical University, Guangzhou 528200, China.

**Keywords:** Skin fibrosis, macrophages, TREM2, single cell RNA sequencing

## Abstract

**Rationale:** Systemic sclerosis (SSc) is a chronic and incurable autoimmune disease with high mortality rates, and skin fibrosis is one of distinguishing hallmarks in the pathogenesis. However, macrophage heterogeneity regulating skin fibrosis remain largely unknown.

**Methods:** We established mouse disease model and performed single-cell RNA-sequencing (scRNA-seq) to resolve the dynamic and heterogenous characteristics of macrophages in skin fibrosis, and the role of TREM2-dependent macrophages in the pathological process was investigated using knockout mice and intraperitoneal transferring TREM2^+^ macrophages combining with functional assays.

**Results:** We show that TREM2-expressing macrophages (TREM2^+^ MФs) accumulate in injured skin of mice treated by bleomycin (BLM) and human SSc, and their gene signatures and functional pathways are identified in the course of disease. Genetic ablation of *Trem2* in mice globally accelerates and aggravates skin fibrosis, whereas transferring TREM2^hi^ macrophages improves and alleviates skin fibrosis. Amazingly, we found that disease-associated TREM2^+^ MФs in skin fibrosis exhibit overlapping signatures with fetal skin counterparts in mice and human to maintain skin homeostasis, but each has merits in skin remodeling and development respectively.

**Conclusion:** This study identifies that TREM2 acts as a functional molecule and a major signaling by which macrophage subpopulations play a protective role against fibrosis, and disease-associated TREM2^+^ MФs in skin fibrosis might undergo a fetal-like reprogramming similar to fetal skin counterparts.

## Introduction

Systemic sclerosis (SSc) is a rare autoimmune disease characterized by vasculopathy, immune dysfunction, and fibrosis of the skin and multiple visceral organs 1, 2. Progressive skin fibrosis is the distinguishing hallmark of SSc, and the degree of skin thickness score correlates with internal organ involvement. Immunological abnormalities contribute to the onset and progression of SSc fibrosis, which is triggered by vascular alternation due to apoptosis and the release of damage-associated molecular patterns (DAMPs) of endothelial cells 3. Previous studies showed that multiple immune cells have been implicated in SSc 4. Macrophages were identified as one of the key drivers of the inflammatory and fibrotic manifestations in SSc, in which macrophages establish a complex communication system with ECM-producing myofibroblasts and endothelial cells via producing pro-fibrotic mediators and various cytokines/chemokines, suggesting their pathogenic role in tissue fibrotic progress 4, 5. Interestingly, macrophages also showed the property of inflammatory resolution via efferocytosis to cleave cellular debris and apoptotic bodies 6, and might have antifibrotic potential 7. Moreover, *in vitro*-trained macrophages were anti-fibrotic in mice and pre-clinical models of SSc 8. These results suggested macrophages are at the crossroads of the SSc pathogenic processes, and might play a bidirectional role in SSc fibrosis. As well known, macrophages are plastic populations and can adopt various activation states depending on their dynamic microenvironment, implying that functionally important cell states are limited in time and space 5, 9. However, macrophage heterogeneity including new cell subpopulations or cell states, in the process of SSc fibrosis remains unclear.

Single-cell RNA-sequencing (scRNA-seq) helps dissect the dynamic process of disease pathology and investigate the transcriptional states of a heterogeneous cell population or cell states at a high resolution 10. In this study, we performed scRNA-seq analysis of skin cells in a mouse disease model to investigate the dynamics features of macrophages. We found skin injury after bleomycin (BLM) treatment massively induced accumulation of monocyte-derived macrophages and a second subpopulation characterized by a high expression of triggering receptors expressed on myeloid cells (*Trem2*). The gene features and functional pathways associated with TREM2^+^ MФs, including phagocytosis, lipid metabolism, angiogenesis, and promoting cell survival were identified during fibrosis. Genetic ablation of *Trem2* in mice globally accelerated and aggravated skin fibrosis, whereas transfer TREM2^hi^ macrophages could improve and alleviate skin fibrosis. Amazingly, TREM2^+^ MФs of skin fibrosis appeared to undergo a fetal-like cellular reprogramming. This phenotype seems to be conserved in human. Overall, these data illustrated the dynamic and heterogenous features of macrophages and identified a therapeutic potentiality of TREM2-dependent macrophages in skin fibrosis, which might provide an opportunity for targeting TREM2 and a novel effective strategy to develop advanced cell therapies for refractory fibrosis of SSc.

## Results

### TREM2^+^ macrophages massively accumulated in skin fibrosis of human and mouse

**Figure 1:**
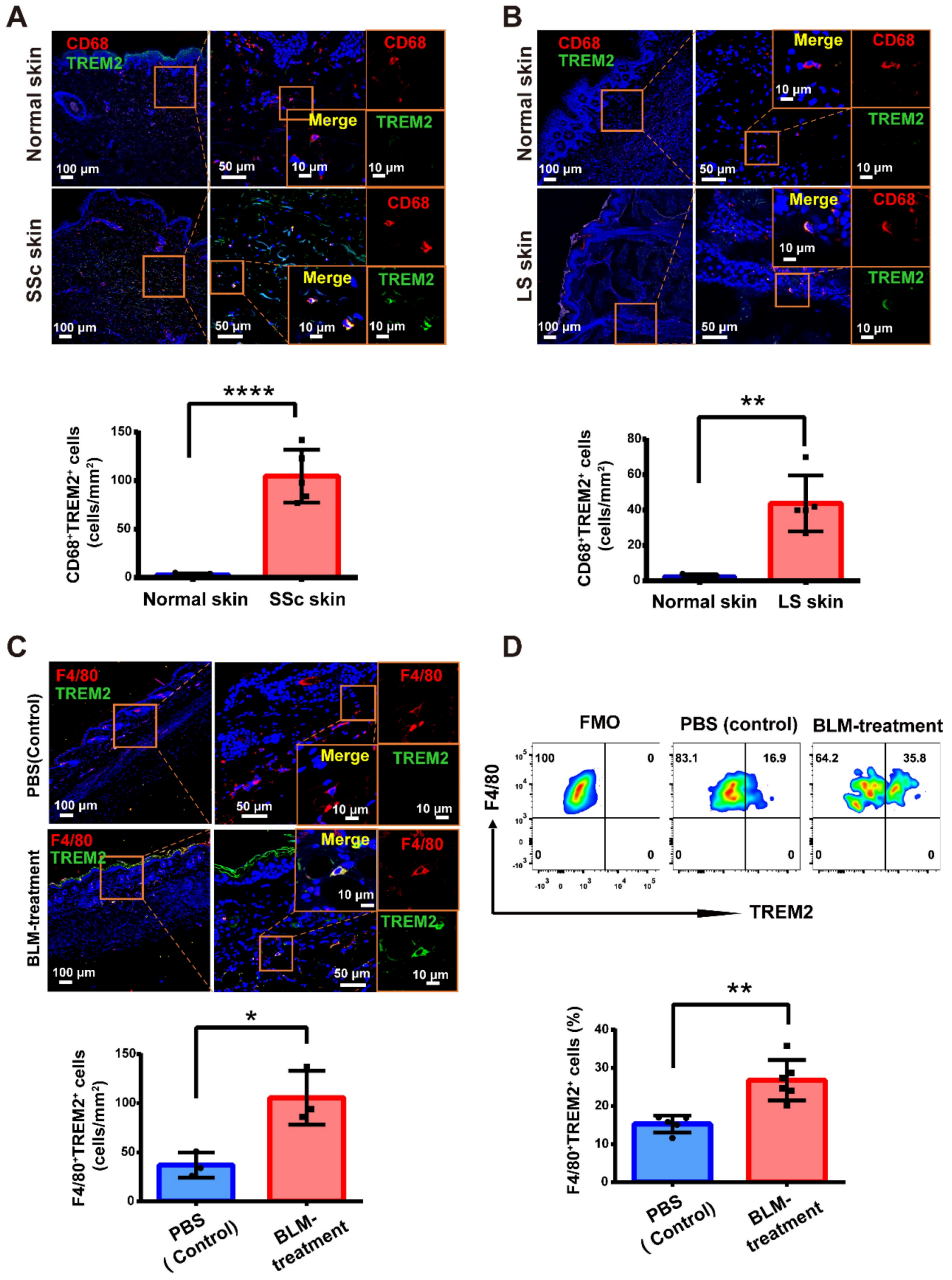
** TREM2^+^ macrophages were dramatically increased in skin fibrosis. (A-B)** Immunofluorescence for CD68 and TREM2 in skin tissue from healthy and patients with skin fibrosis (SSc or LS patients). Scale bars: 100 μm (left panel), 50 μm (middle panel, [bar in insert: 10 μm]), 10 μm (right panel). Representative images of n = 5. **(C)** Immunofluorescence labeling for F4/80 and TREM2 in skin tissue treated by BLM or PBS. Scale bars: 100 μm (left panel), 50 μm (middle panel, [bar in insert: 10 μm]), 10 μm (right panel). Representative images of n = 3 per group. **(D)** Representative FACS plots of TREM2 on F4/80^+^ macrophages (top panel), and the frequency of F4/80^+^TREM2^+^ macrophages after BLM or PBS treatment was shown in the bottom panel. Representative images of n = 5∼6 mice per group.

Previous studies have identified monocytes/macrophages heterogeneity and acted as crucial drivers of the inflammatory and fibrosis in the process of SSc 5. Recent discoveries have turned attention towards the central role of TREM2 receptor in diverse pathologies as a major pathology-induced immune signaling hub for sensing tissue damage 11. Here, immunofluorescence was performed to examine TREM2^+^ MФs alteration in human skin lesions of diffuse cutaneous SSc (dcSSC) and localized scleroderma (LS), and found that there was a dramatic upregulation of CD68^+^TREM2^+^ MФs during skin fibrosis progression (**Figure 1A-B**). Meanwhile, we also observed a significant increase of F4/80^+^TREM2^+^ MФs in mouse skin fibrosis induced by BLM treatment by immunofluorescence staining (**Supplementary Figure S1 and Figure 1C**) and FACS analysis (**Figure 1D**). These data demonstrated that *Trem2*-expressing macrophage subpopulation massively accumulated in injured skin, which might be involved in the pathogenesis of skin fibrosis.

### The temporal dynamics of TREM2^+^ macrophages in skin fibrosis

**Figure 2:**
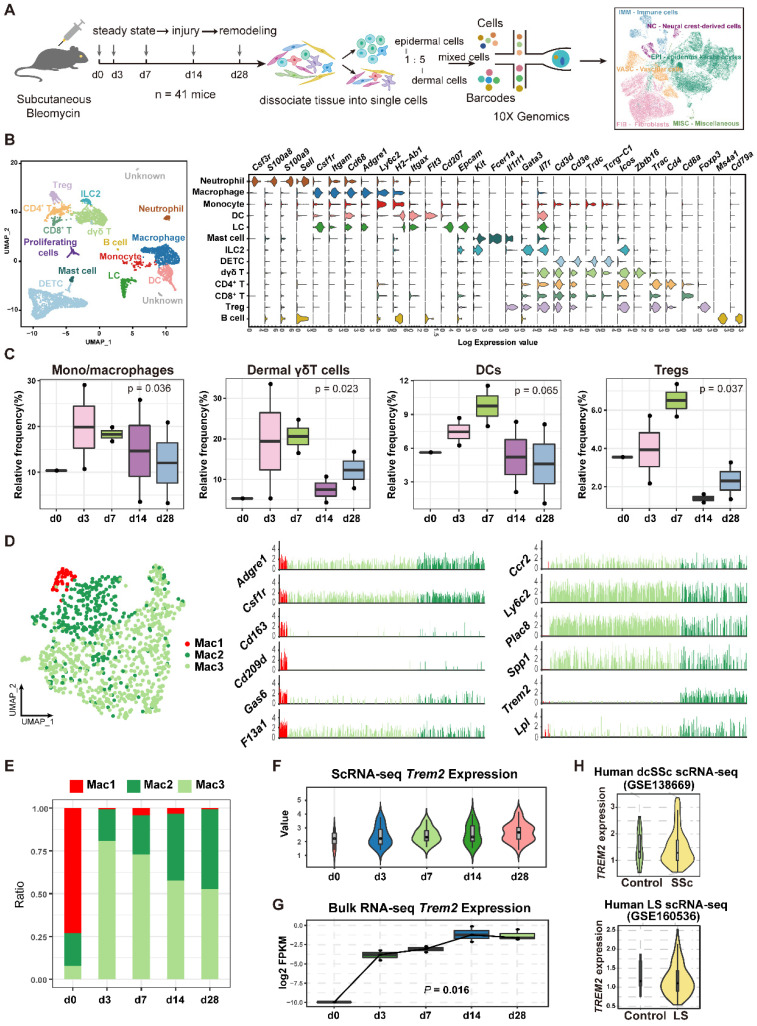
** Dynamic analysis of TREM2^+^ macrophages during mouse skin fibrosis. (A)** Graphical workflow for scRNA-seq analysis. After BLM treatment for 3, 7, 14, and 28 days, epidermal and dermal cells of mouse skin were sorted and mixed on a 1:5 scale, and cDNA libraries were generated with the 10X Genomics platform. n = 3∼5 mice per time point, and d3, d7, d14, and d28 repeated once respectively. **(B)** UMAP visualization of immune cells showing 13 clusters. Each dot represents a cell colored according to the cell cluster (left panel). Gene expression in each cell cluster is shown as a violin plot (right panel). **(C)** Relative frequency of mono/macrophages, dermal γδT cells, DCs, and Tregs at the indicated time points in skin fibrosis. The boxes represent the interquartile range, the horizontal line in the box is the median, and the whiskers represent 1.5 times the interquartile range. Statistics: propeller-sc test, *P* values displayed on plots. **(D)** UMAP projection of macrophage clusters. Each dot represents a cell colored according to the cell cluster (left panel). Log2 average UMI count of selected genes across meta cells of the macrophage populations (middle and right panel). **(E)** Frequencies of macrophage subsets in skin fibrosis (represented by the same colors as in D). **(F)** The expression value of the *Trem2* gene in macrophages is shown as violin plots. The width of each violin reflects the number of cells. **(G)** Relative expression of *Trem2* gene in skin tissue from BLM-treated murine models analyzed by bulk RNA-seq. Statistics: Anova test, *P* values displayed on plots. **(H)** Violin graph showing the expression level of the *TREM2* gene in human macrophages between healthy controls and skin fibrosis patients based on data from dcSSc of Xue et al. (GSE138669) or LS of Mirizio et al. (GSE160536). The width of each violin reflects the number of cells.

To characterize the cellular dynamics during skin fibrosis induced by BLM, we collected skin single-cell suspensions for scRNA-seq after BLM treatment (d3, 7, 14, and 28) and untreated steady state (d0) (**Figure 2A**). After quality control and doublets exclusion (**Supplementary Table S1**), the clusters were categorized into five major cellular compartments based on canonical marker expression: epidermis keratinocytes (*Epcam*), fibroblast-like cells (*Col3a1*), neural crest-derived cells (*Plp1*), vascular cells (*Pecam1*), and immune cells (*Ptprc*) except for miscellaneous cells including skeletal muscle and red blood cells (**Supplementary Figure S2A and Table S2**).

To identify key factors in immune remodeling during skin fibrosis induced by BLM, we further performed immune cell composition analysis from CD45^+^ cells. Here, immune cells were classified into 13 cell types according to the expression levels of the variable genes, and we used the differentially genes to annotate broad myeloid and lymphoid cell populations (**Figure 2B and Supplementary Table S3**). The most prominent changes included an expansion of mono/macrophages, dermal γδT cells, DCs, and Tregs at early stage (d3 or/and d7) after BLM-treatment (**Figure 2C, Supplementary Table S4**).

Macrophages, one of the most significant cell population changes during skin fibrosis induced by BLM, were further repartitioned into three subgroups combined with canonical marker *Csf1r* and *Adgre1*(F4/80). One macrophage population (Mac1) showed enrichment of *Cd163*, *Cd209d*, and *F13a1*, a signature of resident-like macrophages 12. Two additional macrophage populations (Mac2 and Mac3) expressed higher level of* Ccr2*, *Ly6c2*, and *Plac8* genes compared to Mac1, indicating these cells originated from circulation. Mac2 was characterized by a strong expression of *Trem2* gene compared to Mac3 and Mac1, similar to the recently described TREM2^+^ macrophages in diverse pathologies 11, whereas Mac3 (*Ly6c2^+^Trem2^-^*) was thought to have canonical features of mono-macrophages lack of *Trem2* expression (**Figure 2D**). There was a dramatic increase of TREM2^+^ Mac2 during fibrosis progression (**Figure 2E, Supplementary Table S5**), which was also evaluated and reflected by the width of each violin (**Figure 2F**). This result was likewise verified by bulk RNA sequencing, demonstrated by the increased number of *Trem2*-expressing cells (**Figure 2G**). We also identified increased TREM2^+^ MФs in human skin fibrosis from two datasets (GSE138669 and GSE160536) 13, 14 (10X Genomics) (**Figure 2H and Supplementary Figure S2B**). Significantly, *Trem2* was mainly expressed in macrophage subpopulation but not in other cell types from mouse injured skin (**Supplementary Figure S2C**), consistent with *Trem2* expression mainly restricted to mono/macrophages as reported studies 15, 16.

### The transcriptomic characteristics of TREM2^+^ macrophages in skin fibrosis

**Figure 3:**
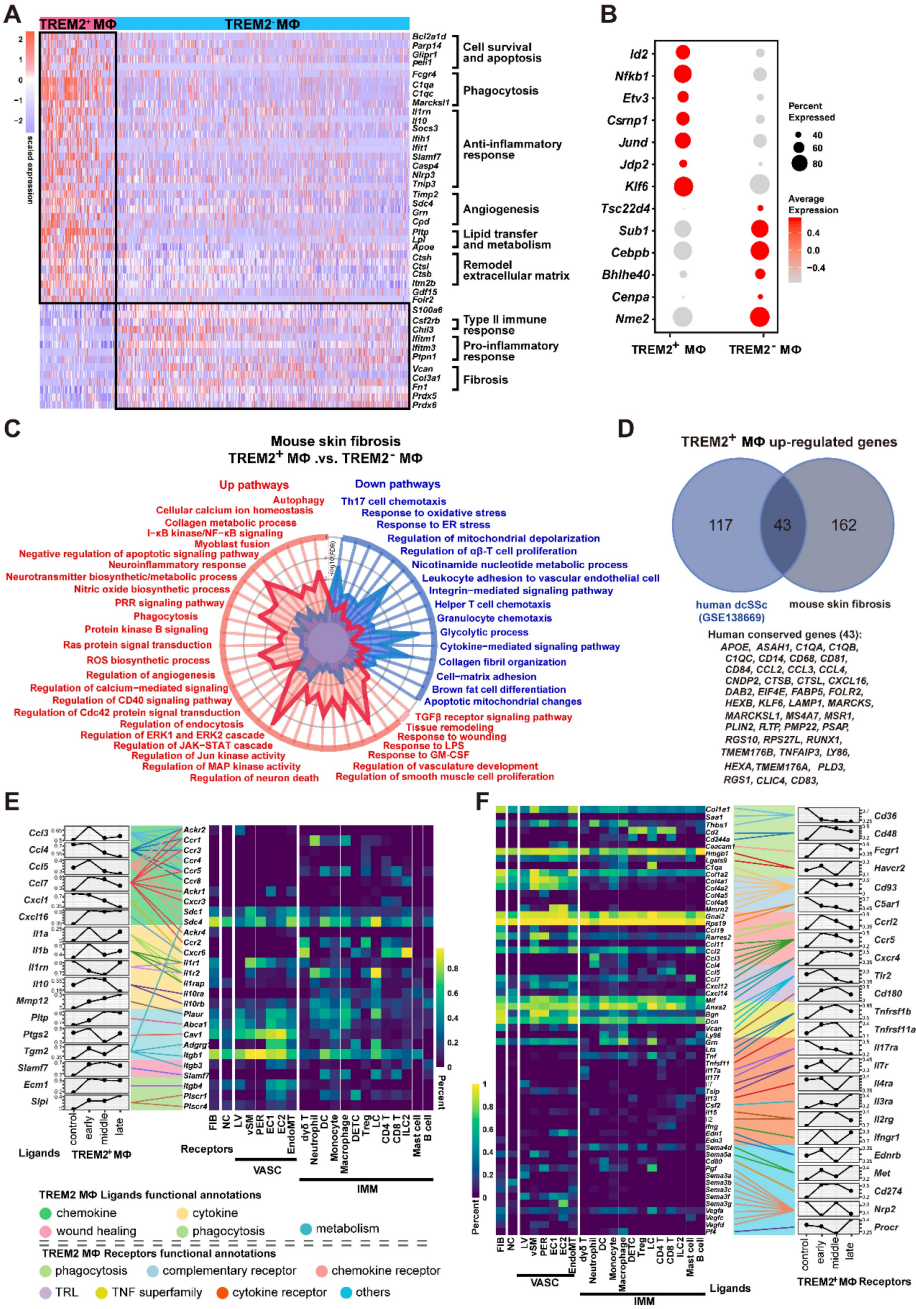
** Analysis of TREM2^+^ MФs features in mouse model. (A)** Heatmap showing the patterns of gene expression for TREM2^+^ and TREM2^-^ MФ. **(B)** Dot plot showing differential expression of transcription factors in TREM2^+^ MФ and TREM2^-^ MФ. Gene expression frequency is indicated by spot size, and expression level is indicated by color intensity. **(C)** A radar map shows pathways associated with TREM2^+^ MФ or TREM2^-^ MФ signatures in mouse skin fibrosis induced by BLM. Statistics: *P* values were calculated with Fisher's exact test (R package clusterprofile), the FDR value displayed on plots which were calculated with Benjamini-Hochberg correction. **(D)** Venn diagram showing common and unique DEGs upregulated in TREM2^+^ MФ from human dcSSc scRNA-seq (GSE138669) and mouse skin fibrosis scRNA-seq induced by BLM. The 43 conserved genes were listed. Statistics: DEGs were identified with R package Seurat (Wilcoxon rank sum test). adjusted *P* value < 0.05 was considered significant. **(E)** E-F. Selected ligand-receptor (L-R) pairs between the TREM2^+^ MФ and other skin cells in the course of skin fibrosis. L-R pairs are grouped functional annotations. Ligands (TREM2^+^ MФ Ligands in E) and receptors (TREM2^+^ MФ Receptors in F) showing significant expression altered throughout skin fibrosis in TREM2^+^ MФ cells. Heatmap shows gene expression frequency of the corresponding receptors or ligands in different cell types. Statistics: Mann-Whitney test, FDR adjusted *P* < 0.05.

Given its dramatic upregulation after skin was injured by BLM, we next sought functional characteristics of TREM2^+^ MФs during skin fibrosis. Differentially expressed gene (DEG) analysis demonstrated significant changes of TREM2^+^ MФs compared with TREM2^-^ MФs, such as a reduction of several genes, including *Chil3* associated with type II immune response, *Ifitm* and *Ptpn1* related to inflammation, as well as *Vcan*, *Col3a1*, *Fn1*, and *S100a4* connected to fibrosis. However, more genes were upregulated in TREM2^+^ MФs, including phagocytosis-associated genes *C1qa* and *Fcgrt*, and angiogenesis and extracellular matrix remodeling related genes *Timp2*, *Sdc4*, *Ctsh*, and *Itm2b* (**Figure 3A, Supplementary Table S6**). Additionally, cell survival genes *Bcl2a1d* and *Igf1*, and lipid metabolism connected with genes* Pltp*,* Lpl* and *Apoe*, were also highly expressed in TREM2^+^ MФs, which was corresponding to previous study 16. Further analysis found that upregulated expression of transcription factors (TFs) genes such as *Jun* and *Klf6* in TREM2^+^ MФs, which might be involved in driving these cells regeneration (**Figure 3B**).

Next, to explore the pathways activated in disease-associated TREM2^+^ MФs, we ranked all genes by their relative expression change in the *Trem2*-expressing subsets, followed by functional classification of the most characteristic genes by gene set enrichment analysis (GSEA). This approach revealed a stronger enrichment of TREM2^+^ MФs-expressed genes (adjusted *P* value < 0.05) compared to TREM2^-^ MФs in pathways related to phagocytosis, angiogenesis, and anti-inflammation, whereas expressed lower number of genes linked with cell-matrix adhesion and immune cell chemotaxis (**Figure 3C, Supplementary Table S7**). This result was in line with the features of TREM2^+^ MФs in Alzheimer's disease (AD) and obesity-related metabolic syndrome 7, 11, suggesting their potential pro-resolving property in skin fibrosis. Meanwhile, we observed remarkable overlap of TREM2^+^ MФs transcriptomic signature between mouse skin fibrosis and human dcSSc. Forty-three of one hundred and sixty genes contained in TREM2^+^ MФs -conserved signature of human dcSSC (*APOE*, *C1QA*, *CSTB*,* CXCL16*, *KLF6*, *LY86*, *FABP5*, and *MSR1* et al.) were also expressed by TREM2^+^ MФs from mouse skin fibrosis (**Figure 3D, Supplementary Table S8**), and there was a substantial overlay including up-regulatory and down-regulatory pathways in TREM2^+^ MФ between mouse skin fibrosis and human dcSSc (pathways highlighted in black** Supplementary Figure S3**). Thus, we may consider TREM2^+^ MФs in human skin fibrosis somewhat resemble to murine TREM2^+^ MФs after BLM treatment in transcriptional features.

Examining L-R pairs in single-cell maps contributes to revealing potential cellular components shaping tissue fate 17. Here, we focused on L-R pairs whose ligand or receptor expression was significantly perturbed during BLM-treated skin (Mann-Whitney test, FDR adjusted *P* < 0.05). Grouping the L-R pairs by functional annotations revealed that receptors of vasculature, immune cells, and fibroblast recognized the upregulated ligands in TREM2^+^ MФs, which were implicated in angiogenesis, autophagy, and lipid metabolism via COX2 (*Ptgs2*)-CAV1, TGM2-SDC4/ITGB1, PTLP-ABCA1, SLPI-PLSCR1/4 L-R interactions, respectively. However, most chemokines expect CXCL16 in TREM2^+^ MФs were downregulated from d3 after BLM treatment, suggesting a suppressive role in the recruitment of immune cells (**Figure 3E, Supplementary Table S9**). We also detected increased receptors in TREM2^+^ MФs receiving signals from other cells were involved in cell survival, phagocytosis, and calcium homeostasis via TNFR2 (*Tnfrsf11b*)-GRN, MET-SEMA4D/SEMA5A, CD48-CD2/CD244, FCGR1-BGP (*Ceacam1*), CD180-LY86, CXCR4-CXCL12, IL4RA-IL13 R-L interactions; and decreased receptors resulting in the dysregulation of coagulation and complement activation corresponding to fibrosis and hypercoagulative state in SSc (e.g., CD36-THBS1/COL1A1/SAA1, IL17RA-IL17A/IL17F, C5AR1-RPS19/GNAIL2) (**Figure 3F, Supplementary Table S9**). Taken together, TREM2^+^ MФs intensively interacted with the immune and stromal compartments, further suggesting that TREM2^+^ MФs might be associated with the pathogenesis of skin fibrosis.

### TREM2-dependent macrophages played a suppressive role in BLM-induced skin fibrosis

**Figure 4:**
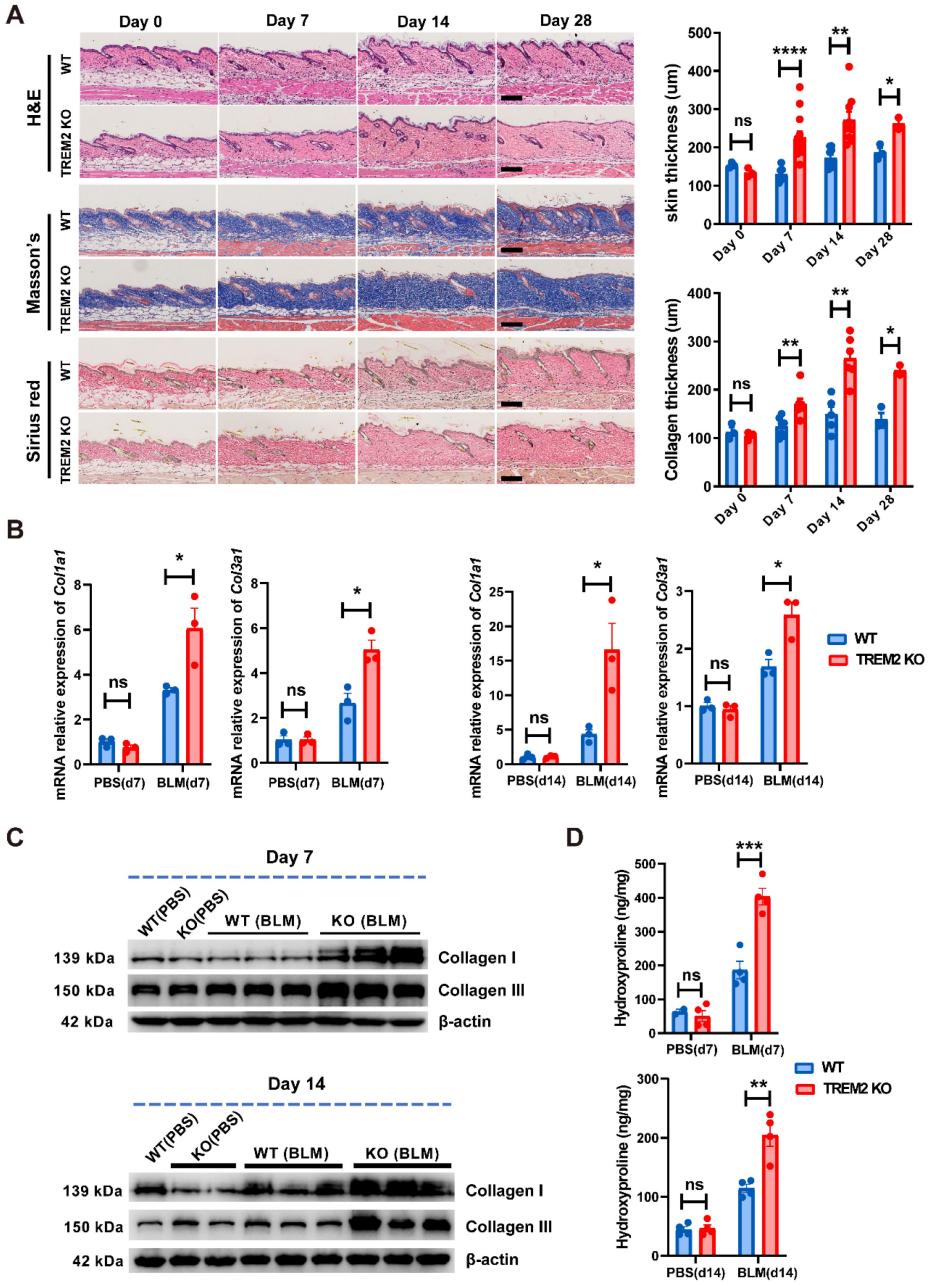
** TREM2 deficiency accelerated and exacerbated skin fibrosis induced by BLM. (A)** Representative H&E, Masson's trichrome, and Sirius Red staining of skin tissue sections after BLM treatment at the indicated time points (left panel). Measurement of skin thickness and collagen thickness in BLM-treated WT and TREM2 KO skin (right panel). The average of skin or collagen thickness values is represented for each sample. **(B)** RT-PCR analyzed the mRNA expression of *Col1a1* and *Col3a1* in skin tissue treated by BLM at the indicated time points. **(C)** Representative western blot assay showing Collagen I (COL1A1) and Collagen III (COL3A1) expression in skin tissue treated by BLM at the indicated time points. **(D)** Quantification of hydroxyproline content were assessed in BLM-treated mouse skin. Images and data shown were representative of at least 3∼5 mice in each time point (every group) corresponding to three independent experiments. Statistics (A-D): Means ± SEM are shown. *P*-values were calculated with the 2-tailed Student's *t*-test, and *P* < 0.05 was considered significant. ns: not significant, *P < 0.05, **P < 0.01).

To determine the function of TREM2 expression in macrophages during skin fibrosis, we treated *Trem2* knockout (*Trem2* KO) mice and littermates (WT mice) with subcutaneous BLM or phosphate-buffered saline (PBS, carrier control) injection every alternate day for 27 days (**Supplementary Figure S4A**). The result showed that compared with WT mice, mice with deletion of *Trem2* showed a significant exacerbation in skin fibrosis on day7 and day14 after BLM treatment, as measured by increased skin thickness using H&E staining and Masson's Trichrome, as well as increased collagen thickness using Sirius red staining (**Figure 4A**). The fibrotic response on day7 and day14 was also demonstrated by collagen deposition in the dermis quantified by qRT-PCR (**Figure 4B**) and western blotting (**Figure 4C**). In addition, Hydroxyproline assay also revealed significantly increased hydroxyproline content in *Trem2* KO mice on day7 and day14 after BLM treatment compared with WT mice (**Figure 4D**). These results indicated *Trem2*-deficiency led to accelerated and aggravated fibrosis in BLM-treated mice.

**Figure 5:**
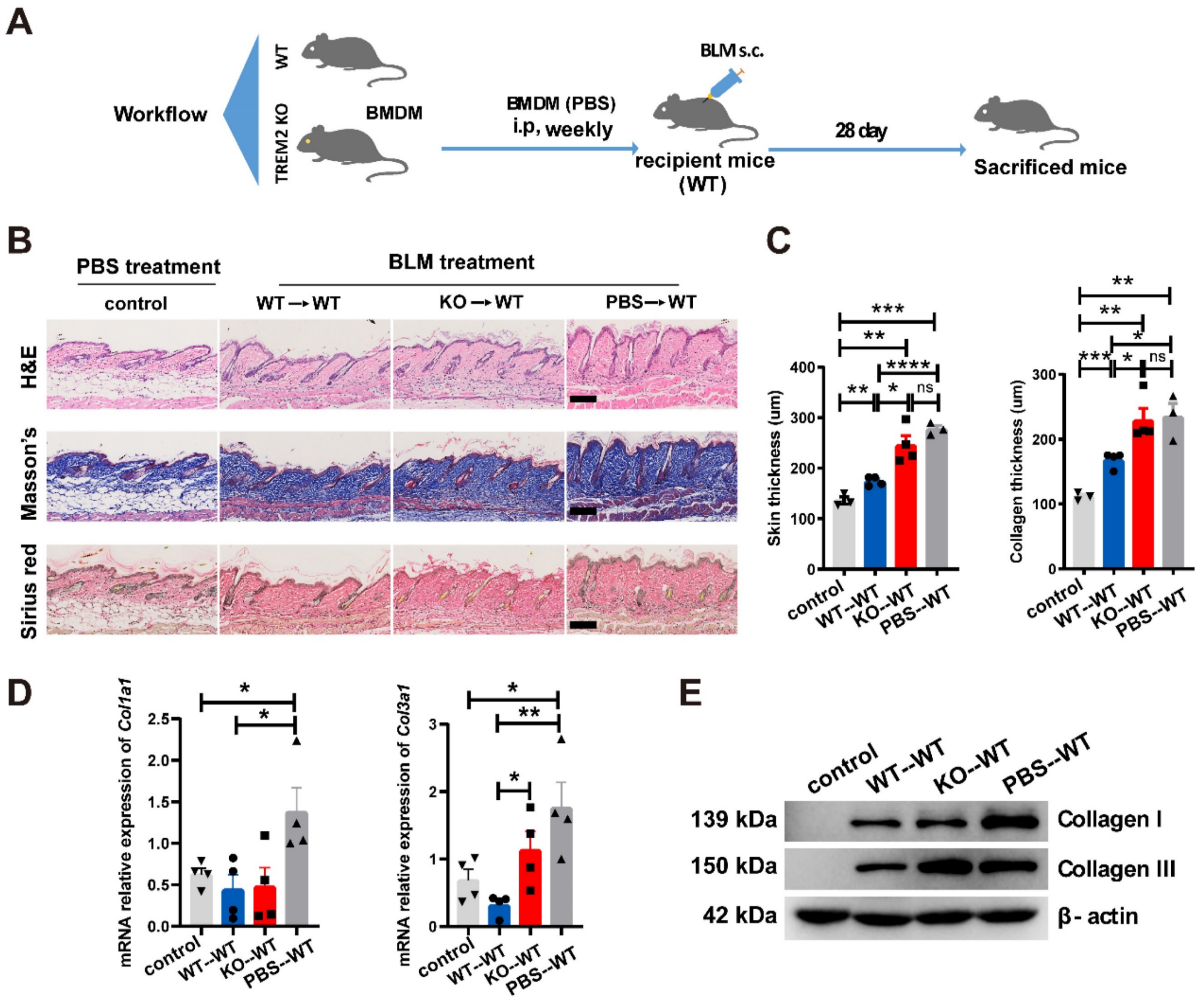
** TREM2^+^ MФs transferring alleviated skin fibrosis induced by BLM. (A)** Schematics for 1 nM VD3-treated BMDM from WT or TREM2 KO mice transferred to recipient mice (WT) treated by BLM for 28 days. **(B-C)** Representative H&E, Masson's trichrome and Sirius Red staining of skin tissue sections after adoptive transfer are shown in B, and skin thickness and collagen thickness are measured as shown in C. The average of nine skin or collagen thickness values is represented for each sample. **(D)** RT-PCR analyzed the mRNA expression of *Col1a1* and *Col3a1* in skin tissue after adoptive transfer from (B-C). **(E)** Representative western blot assay showing Collagen I (COL1A1) and Collagen III (COL3A1) expression in skin tissue after adoptive transfer from (B-C). Images and data shown were representative of at least 3-5 mice in each time point (every group) corresponding to three independent experiments. Statistics, Means ± SEM are shown. *P*-values were calculated with the 2-tailed Student's *t*-test, and *P* < 0.05 was considered significant. ns: not significant, *P < 0.05, **P < 0.01).

To further confirm the suppressive role of TREM2-dependent macrophages in skin fibrosis, bone marrow-derived macrophages (BMDMs) from *Trem2* KO and WT mice were cultured, and then stimulated with 1,25-dihydroxycholecalciferol (active form of VD, VD3) as previous study 18 (**Supplementary Figure S4B and S4C**). *Trem2* expression in cultured WT macrophages treated with VD3 was significantly increased compared to no treatment with VD3, and was lost in *Trem2*-deficient BMDMs in the presence and absence of VD3 (**Supplementary Figure S4D**). Next, BMDMs from *Trem2* KO or WT mice after treatment with VD3 were intraperitoneally injected into WT mice with BLM treatment (recipient mice) weekly and sacrificed mice after 4 weeks (**Figure 5A**). We found that injection of TREM2^hi^ macrophages significantly alleviated the thickness of skin and collagen (**Figure 5B-C**). Moreover, the mRNA expression of *Col1a1* and *Col3a1* (**Figure 5D**) and their protein levels (**Figure 5E**) were significantly decreased compared with that injection of *Trem2*-deficient macrophages. To our knowledge, these results revealed that macrophage subpopulation expressing *Trem2* mitigate fibrosis in a TREM2-dependent manner.

### TREM2^+^ MФs in skin fibrosis exhibit overlapping signatures with fetal skin conterparts in mice and human

**Figure 6:**
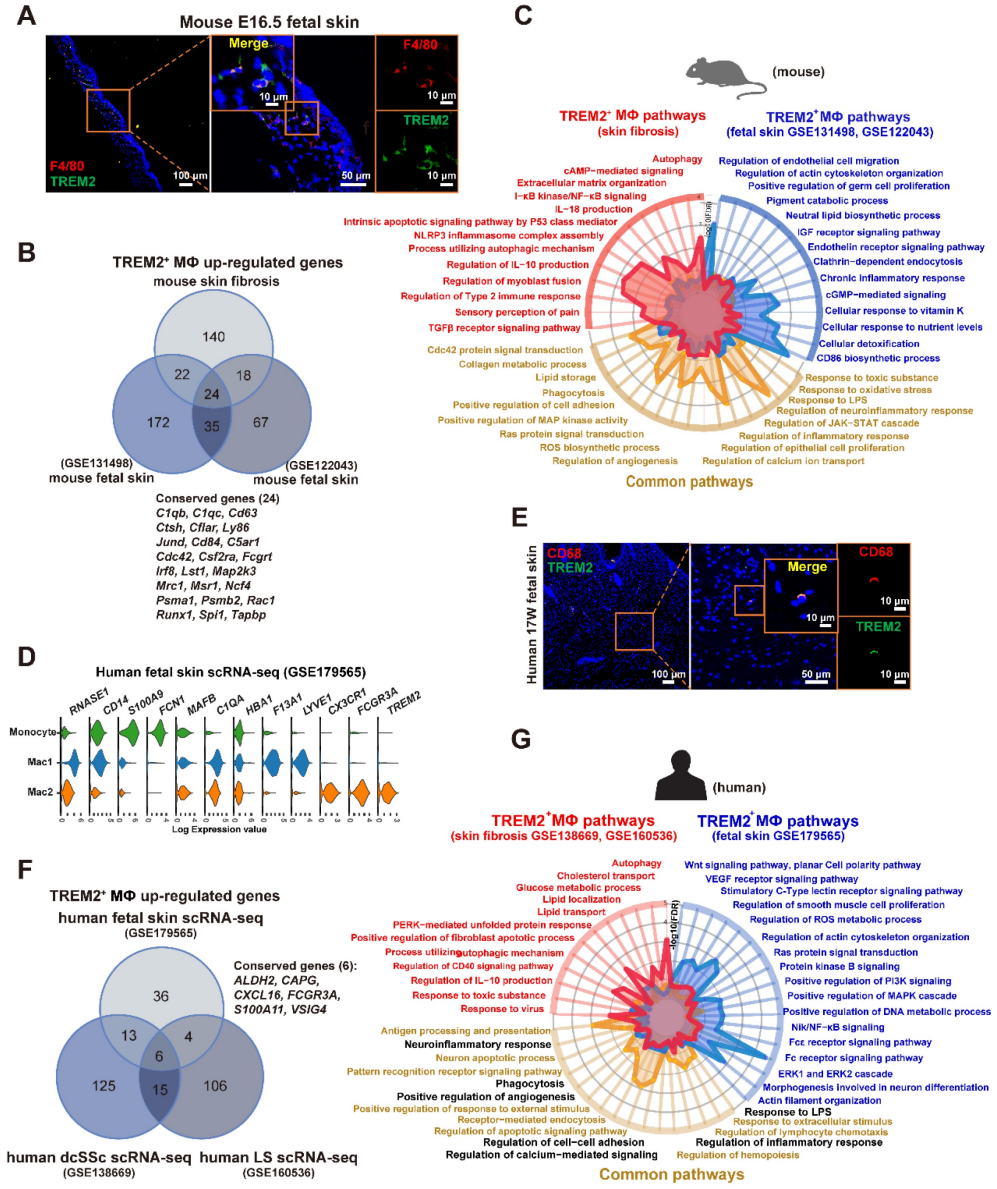
** TREM2^+^ macrophage equivalents in skin fibrosis are present in fetal skin of mouse and human. (A)** Immunofluorescence for F4/80 and TREM2 in mouse E16.5 fetal skin. Scale bars: 100 μm (left panel), 50 μm (middle panel, [bar in insert: 10 μm]), 10 μm (right panel). Representative images of n=3. **(B)** Venn diagram showing common and unique DEGs upregulated in TREM2^+^ MФ from mouse skin fibrosis scRNA-seq, mouse fetal skin scRNA-seq (GSE131498), and mouse fetal skin scRNA-seq (GSE122043) datasets. The 24 conserved genes were listed. Statistics: empirical *P* value was determined to be 0.0076 with permutation test. **(C)** A radar map shows pathways associated with TREM2^+^ MФ signatures in mouse skin fibrosis induced by BLM and/or mouse fetal skin (combined GSE122043 and GSE131498). Statistics: the *P* value were calculated with Fisher's exact test (R package clusterprofile), the FDR value displayed on plots which were calculated with Benjamini-Hochberg correction. **(D)** Violin plot showing gene expression in monocytes, Mac1, and Mac2 according to the human fetal skin scRNA-seq data from Xu et al. (GSE179565). **(E)** Immunofluorescence for CD68 and TREM2 in human fetal skin at 17 weeks estimated gestational age. Scale bars: 100 μm (left panel), 50 μm (middle panel, [bar in insert: 10 μm]), 10 μm (right panel). Representative images of n = 3 (15-17-week fetal skin). **(F)** Venn diagram showing common and unique DEGs upregulated in TREM2^+^ MФ from human fetal skin (GSE179565), human dcSSc (GSE138669), and human LS (GSE160536). The 6 conserved genes were listed. Statistics: DEGs were identified with R package Seurat (Wilcoxon rank sum test), adjusted *P* value < 0.05 was considered significant. **(G)** A radar map shows pathways associated with TREM2^+^ MФ in human skin fibrosis (combined GSE138669 and GSE160536) and/or TREM2^+^ MФ in human fetal skin (GSE179565). Common pathways corresponding with mouse TREM2^+^ MФs were highlighted in black. Statistics: the *P* value were calculated with Fisher's exact test (R pacakge clusterprofile), the FDR value displayed on plots which were calculated with Benjamini-Hochberg correction.

Previous study showed a reemergence of prenatal macrophage cellular programs in lesional skin, such as atopic dermatitis and psoriasis 19, and in brain of AD 20. Interestingly, we found the TREM2^+^ MФ counterpart in mouse fetal skin from datasets (GSE122043, GSE131498) (**Supplementary Figure S5A**), and validated the cell population by immunofluorescence staining (**Figure 6A**). Further analysis showed 24 genes (*C1qb*, *Ly63*, *Lst1*, *Msr1*, *Runx1*, *Spi1 et al*.) in TREM2^+^ MФ-conserved signature from BLM-treated skin were also expressed by the equivalent compartment from mouse fetal skin (**Figure 6B, Supplementary Table S10**), which was much more than TREM2^-^ MФs (4 genes) (**Supplementary Figure S5B, Supplementary Table S11**). We further identified that the observed overlap of 24 genes among upregulated genes in TREM2^+^ MФs from the mouse fetal skin and BLM-treated skin dataset was not a product of random chance via permutation test (*p* < 0.05) (**Supplementary Figure S5C**). Meanwhile, scRNA-seq integration analysis and hierarchical clustering revealed more similar gene signatures of TREM2^+^ MФs between skin fibrosis and fetal skin, compared to that of TREM2^-^ MФs (**Supplementary Figure S5D-E**). The next step is to explore the parallels and discrepancies between these two macrophage subpopulations, which exhibit a similar gene expression pattern yet differ greatly in their environments. We performed a comparative pathway analysis of DEGs in TREM2^+^ MФs between BLM-treated skin and mouse fetal skin, and found that both expressed a high number of genes associated with phagocytosis, angiogenesis, collagen metabolic process, and lipid storage et al. Thus, the TREM2^+^ MФ-conserved transcriptional signature during skin fibrosis closely resembled the counterparts in healthy fetal skin, suggesting that TREM2^+^ MФs in skin fibrosis appeared to undergo a fetal-like cellular reprogramming in mice. However, TREM2^+^ MФs from BLM-treated skin exclusively expressed genes implicated in anti-inflammation and regulation of myoblast fusion in the context of skin fibrosis, which might be involved in aiming to recuperate skin homeostasis; whereas fetal skin TREM2^+^ MФs, in contrast, appearing naturally during embryogenesis alone express genes linked with cellular response to nutrient levels and pigment catabolic process associated with skin development (**Figure 6C, Supplementary Table S12**).

To identify whether human SSc TREM2^+^ MФs present in the normally-development skin, we analyzed and found TREM2^+^ MФ subset in human developing embryonic skin (**Figure 6D**) from our recent work (GSE179565) 21, and verified it using immunofluorescence (**Figure 6E**). Further analysis showed 6 genes (*ALDH2*,* CAPG*,* CXCL16*,* FCGR3A*, *S100A11*,* and VSIG4*) contained in TREM2^+^ MФ-conserved signature from human dcSSC and human LS were also expressed by the equivalent compartment from human healthy fetal skin (**Figure 6F, Supplementary Table S13**).

Hierarchical clustering also revealed more similar gene signatures of TREM2^+^ MФs in skin fibrosis, especially in human dcSSC with fetal skin, compared to TREM2^-^ MФs (**Supplementary Figure S5F**). We also performed a comparative pathway analysis of DEGs among fetal TREM2^+^ MФs, dcSSc TREM2^+^ MФs, and LS TREM2^+^ MФs. TREM2^+^ MФs of fetal skin and pathological states in human expressed many genes associated with phagocytosis, regulation of cell apoptosis, and inflammatory response, et al., these observations paralleled those in mouse TREM2^+^ MФs (pathways highlighted in black **Figure 6G, Supplementary Table S14**). However, human fetal TREM2^+^ MФs exclusively expressed genes implicated in DNA metabolic process and cytoskeleton organization, whereas pathological TREM2^+^ MФs alone express genes linked with autophagy, clearance, and lipid storage (**Figure 6G**). Together, although TREM2^+^ MФs in developing embryonic skin and pathological states share common gene expression patterns, they have their own unique characteristics.

## Discussion

**Figure 7:**
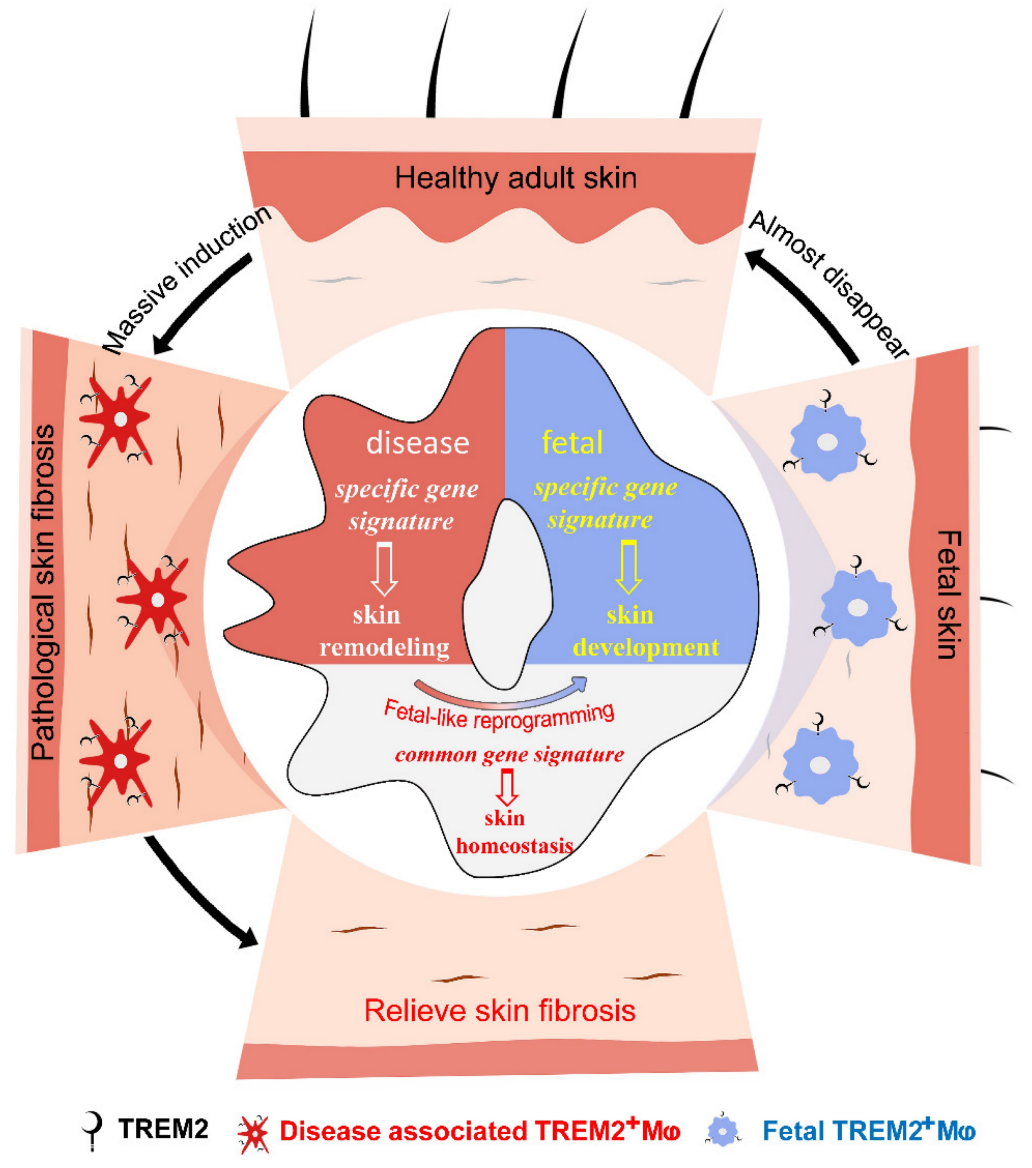
Accumulated TREM2-dependent macrophages in pathology have a fetal-like reprogramming similar to fetal skin counterparts, and play a protective role against skin fibrosis.

We attempted to seek new cell states or subpopulations of macrophages to identify their function in SSc skin fibrosis. Our data supported two conclusions: first, massively aggregated TREM2-dependent macrophages play a protective role during skin fibrosis in a mouse model and human SSc; second, diseases-associated TREM2^+^ MФs in pathological skin fibrosis have a fetal-like reprogramming similar to fetal skin counterparts to maintain skin homeostasis, but each has merits in skin remodeling and development **(Figure 7)**. Altogether, we describe the transcriptional dynamics and characteristics of TREM2^+^ MФs in skin fibrosis at single-cell resolution, and discover their protection against skin fibrosis in a TREM2-dependent manner.

Macrophages were daedal and complex but hitherto underappreciated and poorly understood in the pathogenesis of SSc fibrosis 8. We found that TREM2^+^ MФs were dramatically accumulated in skin fibrosis after BLM treatment and SSc patients (**Figure 1 and Figure 2D-H**), and TREM2 was mainly expressed on monocyte/macrophages in injured skin in line with previous studies 15. Thus, TREM2^+^ MФs might represent a new cell state massively induced by injury signal in skin lesion. Several ligands and receptors in TREM2^+^ MФs were continually downregulated or upregulated in the course of disease, and dynamic crosstalk with other cell types through L-R pairs (**Figure 3E-F**), reflecting their extensive and persistent participation in the pathogenesis of skin fibrosis. We observed that upregulated gene profiles and enriched signaling pathways in TREM2^+^ MФs were associated with phagocytosis, cell survival, angiogenesis, and ECM remodeling, whereas downregulated gene program and signaling pathways were related to inflammation and fibrosis, compared to that of TREM2^-^ MФs (**Figure 3A-C**), which resembled features in the implantation of xenogeneic skin 18. These results suggested TREM2^+^ MФs might play a pro-resolution role in skin fibrosis.

TREM2 acts as a pathology-induced immune signaling hub to sense tissue damage and activate immune modulation. Recent studies demonstrated that macrophages expressing TREM2 were induced in metabolic disease and Alzheimer's disease, in which TREM2 might play a protective function by promoting phagocytosis, blocking inflammation, and regulating lipid metabolism 16, 22, 23. In addition, in the pathogenesis of non-alcoholic steatohepatitis (NASH) and high-fat-diet (HFD)-fed, liver *TIM4^neg^CX3CR1^hi^* macrophages and adipose tissue macrophages (ATMs) have strong similarities with lipid-associated macrophages (LAMs) with increased expression of* Trem2*, *Spp1*, *Cd63*,* Cd9*, and *Gpnmb*, which also share expression of a subset of genes associated with scar-associated macrophages (SAMs) from human cirrhosis (*CD9, CD63, LAGLAS3, SPP1*), and may protect against NASH fibrosis potentially via the containment and/or clearance of dead cells or excess toxic lipids 7. However, CD9^+^TREM2^+^CD63^+^ SAMs expressing Spp1 present in liver and lung fibrosis were presumed to have profibrotic role across species and tissues 24-26. In our study, we demonstrated that TREM2-dependent macrophages might exert a protective role in skin fibrosis via putative capabilities of promoting phagocytosis, angiogenesis, and ECM reconstruction (**Figure 3A-C**), which was further verified in a mouse disease model using conventional knockout mice (**Figure 4**) and TREM2^hi^ macrophages transferring experiments (**Figure 5**). These data suggested that TREM2^+^ macrophage subpopulation might be involved in pathological process of fibrosis with different function although they have similar phenotype.

TREM2-dependent disease-associated microglia (DAM) exhibited similar gene expression patterns typically observed in microglia during embryogenesis 20. Amazingly, we also found TREM2^+^ MФs in mouse fetal skin, and the equivalent compartment in skin fibrosis might undergo a developmental-like transcriptional reprogramming. Although TREM2^+^ MФs in healthy fetal skin and pathological skin fibrosis had some common characterization by endocytosis, angiogenesis, and lipid metabolism, they have their unique features. For example, healthy fetal TREM2^+^ MФs were dominant in cellular responses to nutrient levels aiming at skin development, whereas TREM2^+^ MФs under the pathological state exclusively express gene profiles related to resuming skin homeostasis in mouse and human (**Figure 6**). Nonetheless, further studies will be required to directly assess the roles of TREM2^+^ MФs in embryogenesis and fibrosis-like diseases. In addition, we found TREM2^+^ MФs in human SSc had similar features with that of mouse skin fibrosis after BLM-treatment (**Figure 6**), demonstrating TREM2^+^ MФ signatures appeared to be conserved between the two species, and the mouse model of skin fibrosis induced by BLM treatment might better reflect the pathogenesis of human dcSSc.

A limitation of our study is related to the fact that the detailed mechanisms to activate and regulate TREM2 signaling have yet to be illustrated in the pathogenesis of SSc. Further studies should focus on clarifying how to induce TREM2 signaling in the context of skin fibrosis as well as deciphering how to regulate cell function via downstream molecular pathways in TREM2 engagement and TREM2-dependent manners. In conclusion, we demonstrated massively accumulated TREM2^+^ MФs in skin fibrosis, present their dynamic and characteristics at single-cell transcriptomic landscape, and identified that TREM2 was not only a biomarker of macrophage subpopulation, but also acted as a functional molecule to protect against fibrosis. These data make important contributions to the current understanding of the pathogenesis of SSc fibrosis, especially providing a new perspective on TREM2 as a key target to develop advanced macrophage therapies for fibrosis-like diseases.

## Materials and methods

### Mice

C57BL/6J (WT) was purchased from Guangzhou Ruige Biological Technology Co. Ltd (Guangzhou, China). TREM2 KO mice were obtained from Cyagen company (Suzhou, China). Female mice aged 6-8 weeks and weighed 18-20g were used. All mice were housed and bred in specific pathogen-free conditions at 20-26°C with 30-70% humidity. All animal experiments performed in this study were approved by the Institutional Animal Care and Use Committee of Southern Medical University.

### Quantification and statistical analysis

Statistical analyses were performed using R software 27. Bar plots show the average expression level ± standard error of the mean (SEM) or average ratio ± SEM. *P*-values were calculated with the 2-tailed Student's *t*-test, and *P* < 0.05 was considered significant (*P < 0.05, **P < 0.01, ***P < 0.001). The circos plot, heatmap graph, and Sankey diagram were performed using R packages RCircos, pheatmap, and networkD3, respectively.

### Data and materials availability

All data needed to evaluate the conclusions in the paper are present in the paper and/or the Supplementary Materials. The accession number for the scRNA-seq sequencing data reported in this paper is National Genomics Data Center (NGDC) CRA009260 [https://ngdc.cncb.ac.cn/gsa/browse/CRA009260]. The count matrix data of scRNA-seq have been deposited in Zenodo (https://doi.org/10.5281/zenodo.7455989).

## Supplementary Material

Supplementary material and methods, figures.
