# Supplementary material for: Dynamic pathological analysis reveals a protective role against skin fibrosis for TREM2-dependent macrophages
Source: Theranostics. 2024 Mar 11;14(5):2232–45. doi: 10.7150/thno.94121 (PMC10945340; doi:10.7150/thno.94121)
Supplement: Supplementary file 1 — Supplementary material and methods, figures. [file thnov14p2232s1.pdf]

Dynamic pathological analysis reveals a protective role against skin fibrosis for TREM2-dependent macrophages

Yunsheng Liang [1,2,#], Yongfei Hu [1,#], Jun Zhang [1,#], Haosen Song [1], Xiaoqian Zhang [1], Yishan Chen [1], Yu Peng [1], Lihua Sun [3], Yuzhe Sun [1], Ruzeng Xue [2], Suyun Ji[2], Chuanwei Li [1], Zhili Rong [1], Bin Yang [1,2,*], Yingping Xu [1,*]

[1] Institute of Dermatology and Venereology, Dermatology Hospital, Southern Medical University, Guangzhou 510091, China

[2] Department of Dermatology, Dermatology Hospital, Southern Medical University, Guangzhou 510091, China

[3] Department of Gynaecology and Obstetrics, Nanhai Hospital, Southern Medical University, Guangzhou 528200, China

[#] These authors contributed equally to this work.

Correspondence: Institute of Dermatology and Venereology, Dermatology Hospital, Southren Medical University. E-mail: XuYP01@smu.edu.cn (Y. P. Xu); Yangbin1@smu.edu.cn (B. Yang)

SUPPLEMENTARY MATERIALS AND METHODS

BLM-induced skin fibrosis

Skin fibrosis was induced by BLM treatment, according to a previous study **[1]**. Briefly, female mice aged 6-8 weeks were anesthetized and subcutaneously injected with 100 μl 2 U/ml BLM (Hanhui pharmaceuticals Co. Ltd) on the shaved back at a fixed position every other day for 27 days. The controls were subcutaneously injected with sterilized PBS on the back. Skin tissue samples from the injected position were collected.

Single-cell suspension preparation and FACS analysis

Mice's skin was harvested and incubated with 0.25% Dispase II (04942078001, Roche) for 1-1.5 h at 37°C after removing fat tissue. The separated epidermal and dermal layers were cut into small pieces and digested by 1 mg/ml collagenase IV (40510ES60, Shanghai Yeasen Biotechnology Co.) in RPMI1640 medium (C11875500BT, HyClone), then incubated at 37°C for 2-2.5 h. The suspension was filtered through 70 μm mesh. After washing, the cells were resuspended in 1XPBS for flow cytometry. Living cells of epidermal or dermal suspension were sorted on a FACSAria III flow cytometer (BD Biosciences) using 4', 6-diamidino-2-phenylindole (DAPI) staining. Then sorted epidermal and dermal cells were mixed with a 1:5 ratio for ScRNA-seq analysis.

Skin samples were processed as described above, and dermal cell suspensions were labeled with appropriate antibodies on ice for 30 min. Antibodies included APC-Cy7 anti-mouse CD45

(103116, Biolegend), FITC anti-mouse CD11b (101206, Biolegend), AF700 anti-mouse Ly6C (128024, Biolegend), PE anti-mouse F4/80 (123110, Biolegend), APC anti-mouse TREM2 (FAB17291A, R&D systems). Dead cells were excluded by DAPI staining. Cells were performed on a Celesta cytometer (BD Biosciences) and analyzed using FlowJo v10 (TreeStar).

Bulk RNA sequencing analysis of skin tissue from mouse skin fibrosis

The quality and concentration of RNA were assessed by Agilent Bioanalyzer 2100 and Nanodrop. RNA library preparation and sequencing were performed by Novogene (Beijing, China) on the Illumina NovaSeq 6000 platform. Low-quality sequencing reads were filtered using adapter removal if the read length was < 50 bp and the quality value < 3. The remaining reads were then aligned via STAR v2.7.1a **[2]** using the mouse reference genome (mm10) and GENCODE vM21 annotations. We calculated gene expression levels of all transcripts as fragments per kilobase of exon per million mapped fragments (FPKM) by cufflinks **[3]**. Differentially expressed genes were determined by DESeq2 **[4]** and identified as Benjamini-Hochberg adjusted *P*-value < 0.05. Gene ontology (GO) biological process enrichment analysis was performed using DAVID Bioinformatics Resources 6.8 (https://david.ncifcrf.gov/).

Histology, immunohistochemistry, and Immunofluorescence analysis

Dorsal skin samples from injected sites were collected and fixed with 4% paraformaldehyde for 24 h. The samples were dehydrated in ethanol and embedded in paraffin according to standard protocols. For histologic analysis, paraffin-embedded skin tissues were cut into 3 μm sections, deparaffinized, and stained with hematoxylin-eosin (H&E), Masson's Trichrome kit (BA4079A, Baso Diagnostics Inc. Zhuhai), and Sirius Red Stain kit (G1472, Solarbio life sciences) for morphology and collagen evaluation, following the manufacturer's instructions.

For immunofluorescence labeling, paraffin sections were deparaffinized with Van-Clear solution (Wuhan Huntz Bio-Tech, Wuhan City, China; H-H0102) and rehydrated in 95% ethanol. Antigen retrieval was performed with commercial high-pH repair buffer (Genentech, Shanghai, China; GT102410) in a 95°C water bath for 15 min. Sections were blocked with 10% goat serum or 5% BSA and incubated with primary antibodies for 1 h at 37°C. The primary antibodies for mouse samples were PE anti-mouse F4/80 (clone: BM8, 123110, Biolegend), rat anti-mouse TREM2 (Clone: 237920, MAB17291, R&D systems). The primary antibodies for human samples were mouse anti-human CD68 (clone: PG-M1, IR61361-2A, Agilent/DAKO), rabbit anti-human TREM2 (clone: D8I4C, 91068S, CST). After rinsing with PBS, the slides were incubated for 1 h at 37°C with the following secondary antibodies: goat anti-mouse IgG Alexa Fluor 594 (ZF-0513, ZSGB Biotech), goat anti-rabbit IgG Alexa Fluor 488 (ZF-0511, ZSGB Biotech), and rabbit anti-rat IgG Alexa Fluor 488 (RS3229, ImmunoWay). After three washes with PBS, the sections were counterstained with DAPI (ZLI-9557, ZSGB Biotech) and mounted, and images were acquired with a confocal microscope (Nikon, Tokyo, Japan; Model A1R).

ScRNA-seq library construction and sequencing

Single cells were obtained as gel bead-in-emulsions (GEMs) using the 10X Genomics (Pleasanton, CA, USA) Chromium platform based on the manufacturer's protocol. Briefly, cell suspensions of skin tissue with reverse transcription master mix were loaded onto the 10X Genomics Single Cell 3' chip and partitioned into GEMs with gel beads precoated with oligonucleotides containing a poly-dT primer sequence to capture released mRNAs, a 16-bp 10X barcode for cell indexing, and a 10-bp unique molecular identifier (UMI) to distinguish between transcripts. After reverse transcription, barcoded cDNAs for each sample were amplified to generate a sufficient volume for library construction using the Single Cell 3' Reagent Kit (v3 chemistry). Libraries were subjected to 150-bp paired-end sequencing performed by Novogene (Beijing, China) on a NovaSeq 6000 system (Illumina, San Diego, CA, USA).

Data preprocessing for single-cell gene expression profiling

The Cell Ranger 4.0.0 pipeline (10X Genomics) was used to align and aggregate all reads from scRNA-seq to the mouse (*Mus musculus*) reference genome (mm10) [10x Genomics provides pre-built references for mouse genomes: https://cf.10xgenomics.com/supp/cell-exp/refdata-gex-mm10-2020-A.tar.gz] with default settings and produce a balanced "filtered feature bc matrix".

Cell heterogeneity, clustering, integration, and visualization

The resulting gene–barcode matrix was imported into the SCANPY software (Version 1.7.1) **[5]**, where sample information was added. Subsequently, quality control was conducted to ensure that all samples analyzed contained highly consistent quality. First, we applied quality control to cells according to three different metrics step by step, including the total UMI count, the number of detected genes, and the proportion of mitochondrial gene count per cell. Specifically, we filtered cells with less than 2000 UMI count or 200 detected genes, as well as cells with more than 5% mitochondrial gene count. To remove potential doublets, we also removed cells with the number of detected genes above 6,000. Furthermore, we removed potential doublets predicted by Scrublet **[6]**. Next, after quality control, we applied the library-size correction method to normalize the raw count matrix by using normalize_total function in SCANPY. We employed the workflow of SCANPY to perform dimension reduction and graph-based unsupervised clustering on scRNA-seq data. Briefly, we first selected 2000 highly variable genes (HVGs) for downstream analysis by using scanpy.pp.highly_variable_genes function with parameter “n_top_genes=2000”. Then, effects of the total count per cell and the percentage of mitochondrial gene count were regressed out by using scanpy.pp.regress_out function. LIGER (Version 2.0.1) software **[7]** was used to integrate the different samples and variation in the cells’ transcriptional profile was visualized by uniform manifold approximation and projection for dimension reduction (UMAP) function in SCANPY, and the initial dim number for PCA analysis was set to 30. PCA components with standard deviation > 1.25 (generated by ElbowPlot) and JackStraw score < 0.05 (generated by Seurat R package **[8]** ScoreJackStraw function) were used to generate two-dimensional UMAP. Leiden algorithm **[9]** was

used to identify clusters within cell populations (Leiden clustering resolution = 0.5, n_pcs=30). Next, each sample Scanpy object file was converted to Seurat object file with SeuratDisk R package (https://github.com/mojaveazure/seurat-disk). Differential expressed genes were calculated in Seurat R package using the FindMarkers function, which performed a two-sided Wilcoxon rank sum test restricted to genes expressed in at least 25% of cells in either of the two populations compared, and with a natural log fold change cut-off of 0.25. All p-values were adjusted for multiple testing using the Benjamini-Hochberg method.

To compare with public mouse fetal skin and human SSc scRNA-seq data, public data were downloaded and analysed according the workflow of Seurat R package. Integration analysis between public scRNA-seq data and our mouse scRNA-seq data were performed within R v3.6.2 and with Seurat v 3.1.1**[10]**. Briefly, the 2000 anchors were used to integrate all Seurat objects created above: (i) by finding the integration anchors (Seurat::FindIntegrationAnchors), (ii) by integrating the objects (Seurat::IntegrateData), (iii) using all.genes to scale the data and regress out the nCount_RNA, and (iv) calculating the top 30 PCAs and using them for dimensional reduction and identifying cell clusters by using a resolution of 0.5 and 1. To identify the differentially expressed genes, we used the Seurat::FindMarkers function and compared every period to its corresponding control for every cell type. For visualization of the scRNAseq data the visualization methods in Seurat and ggplot2 R package were implemented. For gene set activate analysis in scRNAseq data, AUCell method from AUCell Bioconductor packages were used to calculate AUCell score **[11]**.

Cell-Cell communication analysis of mouse scRNA-seq data

To investigate cell-cell communication in mouse scRNA-seq data, we inferred cell-cell interactions using the NATMI method **[12]**. NATMI takes a slightly different approach, focusing on learning interactions between cells using bulk or single-cell expression data, not addressing the specifics of how these regulatory interactions impact downstream gene expression. NATMI uses large-scale ligand-receptor databases to create prior knowledge networks. It then calculates a weight for each interaction between genes, based on three expression-based metrics from the dataset of interest, which are used to determine cell type interactions. Meanwhile, NATMI accept mouse scRNA-seq data as input to analyse cell-cell interactions. Briefly, Seurat normalized expression values were converted to CPMs and then grouped by cell subtype and sample prior to NATMI analysis (https://github.com/asrhou/NATMI). The python 3 versions of NATMI ExtractEdges with the suggested dependency versions were run on each sample with default settings. The predicted ligand-receptor interactions were then read into R for further analysis. For a cell-cell connection to be kept for further analysis, the ligand and receptor must be expressed in >10 cells. Connections were also filtered out if receptor or ligand detection rate < 0.2. Cluster autocrine-signaling and interactions where the ligand and receptor were the same genes were removed for data presentation and interpretation. Furthermore, interactions that were not seen in at least two samples were removed.

Gene Set Enrichment Analysis

To investigate biological pathways correlated with the course of skin fibrosis, we performed a GSEA analysis **[13].** The needed cells from each sample were aggregated to create pseudo-bulk counts, then ClusterProfile R package use the pseudo-bulk counts to do GSEA. The reference gene sets of GSEA, which was called as "c2.cp.kegg.v7.4.symbols.gmt, and c5.go.bp.v7.4.symbols.gmt" were downloaded from the Molecular Signatures Database. The threshold was set at $P < 0.05$ and false discovery rate (FDR) $q < 0.1$. Function and pathway enrichment analysis of differential expressed genes were also performed with ClusterProfile.

Public scRNA-seq data processing

Single-cell RNA-seq counts were obtained from the GEO database as the raw expression matrix of unique molecular identifier counts (UMI counts; indicative of the number of unique RNA molecules detected) for each gene in each cell group. UMI counts from each dataset were filtered on the following criteria: cells with > 1000 UMI counts, > 200 features, < 7500 features, and < 25% mitochondrial gene expression, then normalized and log-transformed using the LogNormalize function in the Seurat package. We integrated all the datasets using the 'scTransform' package for batch correction and scRNA-seq data integration. It was then followed by a combined principal component analysis (PCA), computing 50 principal components, of which the first 30 (based on an elbow plot) were used to compute the Uniform Manifold Approximation and Projection (UMAP) analysis for dimensionality reduction. For the differential expression analysis between Trem2 positive cells and Trem2 negative cells, the cutoff values log (Fold Change) and *P*-value were set to 0.3 and 0.05, respectively.

Consistence with publicly available mouse skin single-cell data

In order to demonstrate the consistency of our cell identity assignments with previous efforts in mouse skin single-cell data, we acquired publicly available mouse skin single-cell data(GSE129218) and retained the cell identity definitions used in the prior study. Combining cells of the same type into a pseudo-bulk, we calculated the correlation between cell type pseudo-bulks from our dataset and the publicly available data. The results indicate that our cell identity definitions align consistently with the definitions from the previous study.

Genetic similarity of TREM2$^+$ macrophages between mouse fetal skin and BLM-treated skin

To demonstrate the genetic similarity between TREM2$^+$ macrophages in mouse fetal skin and TREM2$^+$ macrophages in mouse systemic sclerosis (SSc), we employed two distinct datasets of mouse fetal skin single-cell data (GSE122043, GSE131498). We integrated the TREM2$^+$ and TREM2$^-$ macrophages from both datasets with the TREM2$^+$ and TREM2$^-$ macrophages in our dataset. Additionally, we combined cells from the above six different source into individual pseudo-bulks. Subsequently, based on the Euclidean distance metric, we performed hierarchical clustering with "hclust" method in R package stat. The outcomes of this analysis revealed that, compared to

TREM2$^-$ macrophages, TREM2$^+$ macrophages in both mouse fetal skin and mouse SSc exhibit a higher degree of genetic similarity.

In order to demonstrate that the occurrence of an intersection of 24 genes in the overlap of three upregulated gene sets is not a result of random chance, we employed a permutation test. Specifically, within each respective dataset of macrophages scRNA-Seq, we randomly selected the same number of expressed genes as the upregulated genes in Trem2$^+$ macrophages. Subsequently, we calculated the number of genes present in the intersection of the three selected gene sets. This process was repeated 1000 times, and the frequency of occurrences where the intersection size exceeded 24 was computed. The resulting empirical p-value was determined to be 0.0076. As a result, we conclude that the observed overlap of 24 genes among upregulated genes in Trem2$^+$ macrophages from the mouse fetal skin dataset (GSE122043), the mouse skin fibrosis dataset (our data), and the Trem2$^+$ macrophages dataset (GSE131498) is not a product of random chance.

Culture of bone marrow derived macrophage (BMDMs) *in vitro*

According to previously published protocols, BM was isolated and induced into macrophages *in vitro* **[14]**. Briefly, femurs and tibias were removed from WT or TREM2 KO mice after being sacrificed. The bones were immersed in 75% ethanol for 5 min, then put in PBS. BM was collected by flushing with 10 ml PBS. Red blood cells were lysed for 3 min with 1X commercial lysis buffer (00-4300-54, eBioscience). After neutralizing, the cells were filtered through a 70-μm cell strainer (BD Biosciences; Franklin Lakes, NJ, USA; 352350). Cells were washed and resuspended in 10 ml of DMEM medium containing 10% fetal bovine serum, 100 U/ml penicillin, 100 μg/ml streptomycin, and 10 ng/ml murine granulocyte-macrophage colony-stimulating factor (GM-CSF, 315-03, PeproTech). On d 4, 10 ml of complete medium was added to the cultures. On d 7, the cells were stimulated with 1 nM 1α, 25-dihydroxycholecalciferol (D1530, Sigma-Aldrich) for 24 h. Cells were harvested for BMDM injection *in vivo*.

Enzyme-linked immunosorbent assay (ELISA)

50mg skin tissue was collected and rinsed with 1×PBS, homogenized in 500 uL of 1×PBS and stored overnight at -20°C. After two freeze-thaw cycles were performed to break the cell membranes, the homogenates were centrifuged for 5 minutes at 5000×g (RCF), 4°C. The hydroxyproline (HYP) in the supernatant was measured using an ELISA kit (CSB-E08839m, CUSABIO), according to the manufacturer's instructions.

Western blot

Skin tissue samples were harvested and lysed in radioimmunoprecipitation assay buffer (P0013B, Beyotime) supplemented with protease and phosphatase inhibitor cocktail (P1046, Beyotime), and diluted by reducing SDS-PAGE loading buffer (ComWin Biotech, Beijing, China, CW0027). The lysates were resolved by 12% SDS-PAGE and transferred to polyvinylidene difluoride membranes, followed by blocking in 5% skimmed milk for 1 h. The membranes were incubated with primary

antibodies, including rabbit anti-mouse collagen I (1:1000, ab270993, Abcam), rabbit anti-mouse collagen III (1:1000, ab184993, Abcam), and mouse anti-mouse β-actin (1:5000, 66009-1-Ig, Proteintech). HRP-labeled goat anti-mouse IgG (H+L) (Proteintech, SA00001-1, 1:5000) and HRP-labeled goat anti-rabbit IgG (H+L) (Proteintech, SA00001-2, 1:5000) were used as the secondary antibodies. The blots were visualized by enhanced chemiluminescence detection reagents (Merck Millipore, Billerica, MA). Images have been cropped for presentation.

RT-PCR analysis

Skin tissue samples were harvested and homogenized in Trizol reagent (15596-026, Invitrogen). Total RNA was extracted and quantified by NanoDrop ND-1100 (NanoDrop Technologies, Wilmington, DE). The RNA was reverse-transcribed to cDNA with PrimeScript™ RT reagent Kit with gDNA Eraser (RR047A, Takara). Quantitative real-time PCR (qRT-PCR) was performed using TB Green™ Premix Ex Taq™ II (RR820A, Takara) by Real-Time PCR Detection System (Bio-Rad). The used primer sequences were listed as follows: *Trem2* forward 5'-CTTCCTCCTCTTTCCAAGGAATC-3' and reverse 5'-TGCTGGCTGCAAGAAACT-3'; *Col1a1* forward 5'-AGACCTGTGTGTTCCCTACT-3' and reverse 5'-GAATCCATCGGTCATGCTCTC-3'; *Col3a1* forward 5'-GTGACTCAGGATCTGTCCTTTG-3' and reverse 5'-GTAGAAGGCTGTGGGCATATT-3'. All genes were normalized to the expression of β-actin forward 5'- TGTGACGTTGACATCCGTAA-3' and reverse 5'-GCTAGGAGCCAGAGCAGTAA-3'.

SUPPLEMENTARY REFERENCE

1. Cheon SY, Park JH, Ameri AH, Lee RT, Nazarian RM, Demehri S. IL-33/Regulatory T-Cell Axis Suppresses Skin Fibrosis. J Invest Dermatol. 2022; 142: 2668-76 e4.
2. Dobin A, Davis CA, Schlesinger F, Drenkow J, Zaleski C, Jha S, et al. STAR: ultrafast universal RNA-seq aligner. Bioinformatics. 2013; 29: 15-21.
3. Trapnell C, Williams BA, Pertea G, Mortazavi A, Kwan G, van Baren MJ, et al. Transcript assembly and quantification by RNA-Seq reveals unannotated transcripts and isoform switching during cell differentiation. Nat Biotechnol. 2010; 28: 511-5.
4. Love MI, Huber W, Anders S. Moderated estimation of fold change and dispersion for RNA-seq data with DESeq2. Genome Biol. 2014; 15: 550.
5. Wolf FA, Angerer P, Theis FJ. SCANPY: large-scale single-cell gene expression data analysis. Genome Biol. 2018; 19: 15.
6. Wolock SL, Lopez R, Klein AM. Scrublet: Computational Identification of Cell Doublets in Single-Cell Transcriptomic Data. Cell Syst. 2019; 8: 281-91 e9.
7. Welch JD, Kozareva V, Ferreira A, Vanderburg C, Martin C, Macosko EZ. Single-Cell Multi-omic Integration Compares and Contrasts Features of Brain Cell Identity. Cell. 2019; 177: 1873-87 e17.
8. Butler A, Hoffman P, Smibert P, Papalexi E, Satija R. Integrating single-cell transcriptomic data across different conditions, technologies, and species. Nat Biotechnol. 2018; 36: 411-20.
9. Lun AT, Bach K, Marioni JC. Pooling across cells to normalize single-cell RNA sequencing data with many zero counts. Genome Biol. 2016; 17: 75.

10. Stuart T, Butler A, Hoffman P, Hafemeister C, Papalexi E, Mauck WM, 3rd, et al. Comprehensive Integration of Single-Cell Data. Cell. 2019; 177: 1888-902 e21.
11. Aibar S, Gonzalez-Blas CB, Moerman T, Huynh-Thu VA, Imrichova H, Hulselmans G, et al. SCENIC: single-cell regulatory network inference and clustering. Nat Methods. 2017; 14: 1083-6.
12. Hou R, Denisenko E, Ong HT, Ramilowski JA, Forrest ARR. Predicting cell-to-cell communication networks using NATMI. Nat Commun. 2020; 11: 5011.
13. Subramanian A, Tamayo P, Mootha VK, Mukherjee S, Ebert BL, Gillette MA, et al. Gene set enrichment analysis: a knowledge-based approach for interpreting genome-wide expression profiles. Proc Natl Acad Sci U S A. 2005; 102: 15545-50.
14. Henn D, Chen K, Fehlmann T, Trotsyuk AA, Sivaraj D, Maan ZN, et al. Xenogeneic skin transplantation promotes angiogenesis and tissue regeneration through activated Trem2(+) macrophages. Sci Adv. 2021; 7: eabi4528.

SUPPLEMENTARY FIGURES

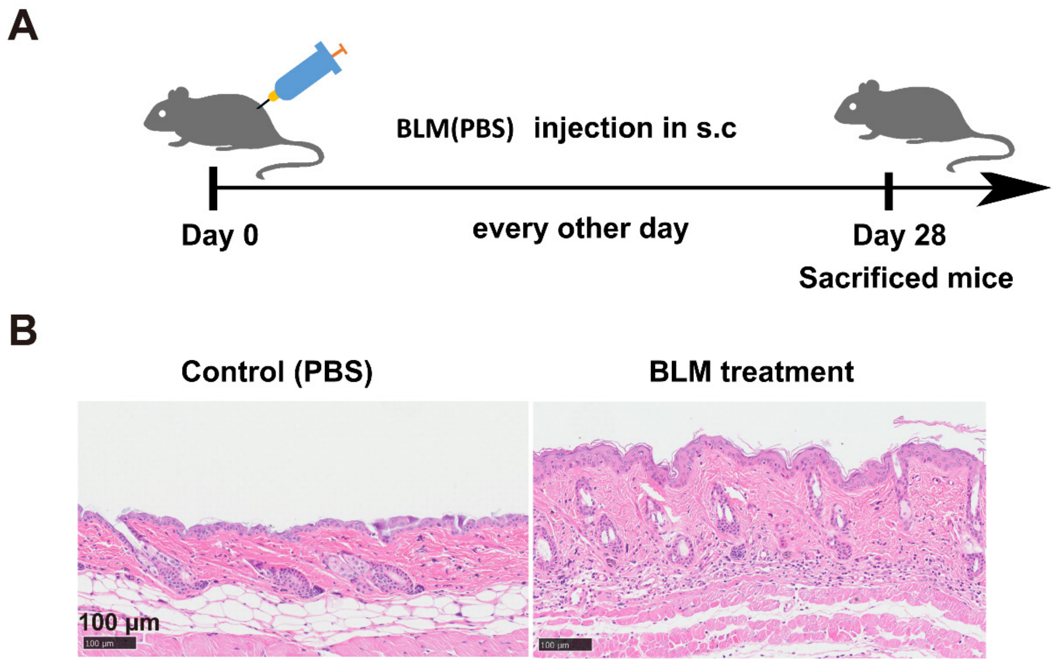

Supplementary Figure S1. The mouse model of skin fibrosis induced by BLM (Related to Figure 1C-D).

A. The mouse model of skin fibrosis was established: mice were treated with subcutaneous BLM or phosphate-buffered saline (PBS, carrier control) injection every alternate day for 27 days, and were sacrificed on day 28.

B. Representative H&E staining of skin tissue sections after BLM treatment in mice.

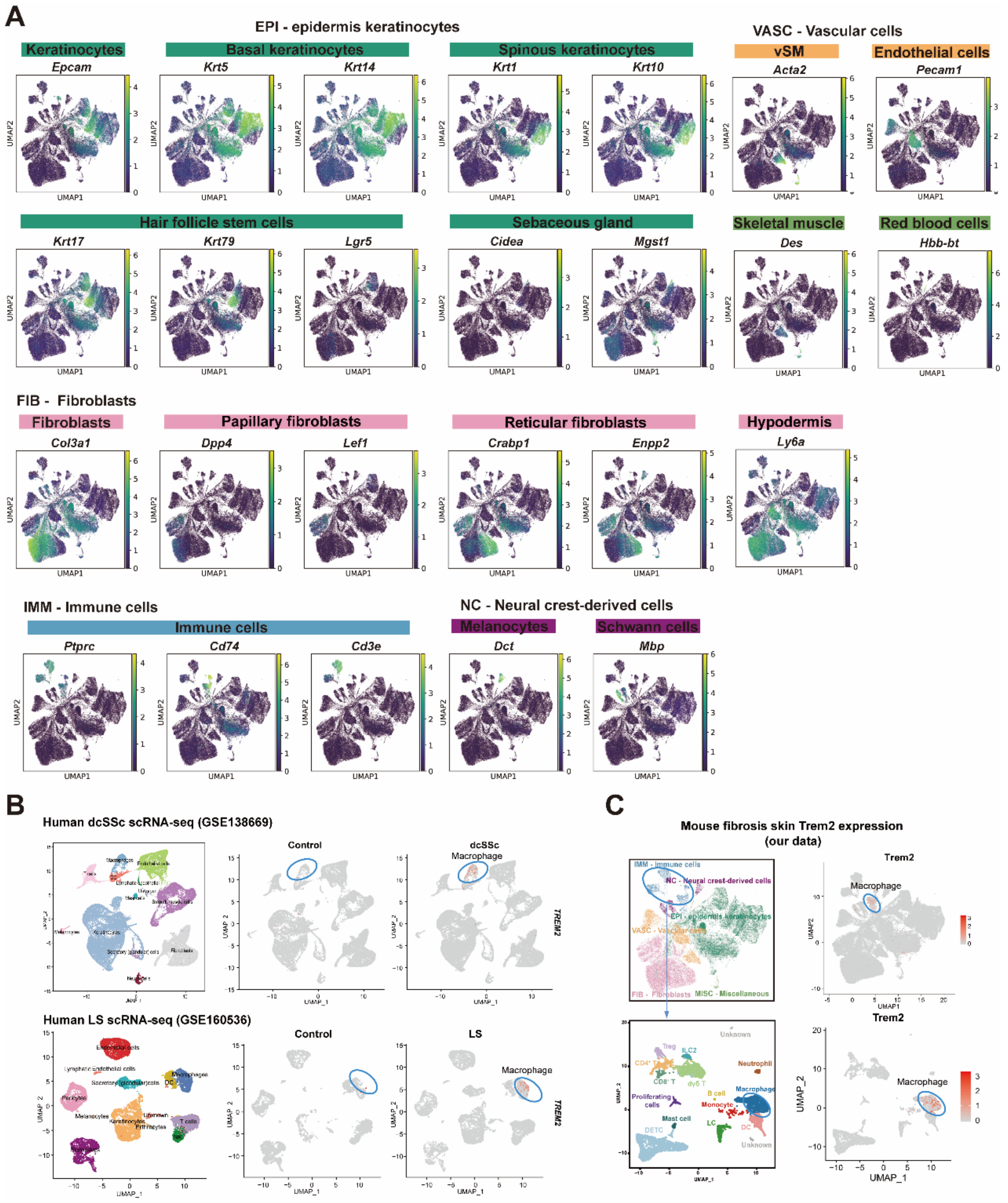

Supplementary Figure S2. Cell subsets in skin fibrosis and *Trem2* expression in cell population were analyzed (Related to Figure 2).

A. UMAP visualization of cells in skin fibrosis for cluster identification. Keratinocytes (*Epcam*) were identified as basal keratinocytes (*Krt5*, *Krt14*), spinous keratinocytes (*Krt1*, *Krt10*), hair follicle stem cells (*Krt17*, *Krt79*, *Lgr5*), and sebaceous gland (*Cidea*, *Mgst1*). Fibroblasts (*Col3a1*) included papillary (*Dpp4*, *Lef1*), reticular (*Crabp1*, *Enpp2*), and hypodermal fibroblasts (*Ly6a*). Other lineages were observed: skeletal muscle cells (*Des*), red blood cells (*Hbb-bt*), melanocytes (*Dct*), Schwann

cells (*Mbp*), immune cells (*Ptprc*, *Cd74*, *Cd3e*), vSM (*Acta2*) and endothelial cells (*Pecam1*). vSM: vascular smooth muscle cells.

B. UMAP visualization shows a re-analysis of the human dcSSc scRNA-seq dataset (GSE138669) and human LS scRNA-seq dataset (GSE160536). Cells are colored by cell type (left panel). The expression of *Trem2* is shown in UMAP (right panel). dcSSc: diffuse cutaneous systemic sclerosis; LS: localized scleroderma.

C. UMAP visualization shows the analysis of mouse skin treated by BLM from our datasets. Cells are colored by cell type, and the expression of *Trem2* is shown in UMAP (upper panel). Meanwhile, UMAP visualization shows a re-cluster of immune cells (blue border), cells are colored by cell type, and the expression of *Trem2* is shown in UMAP (below panel).

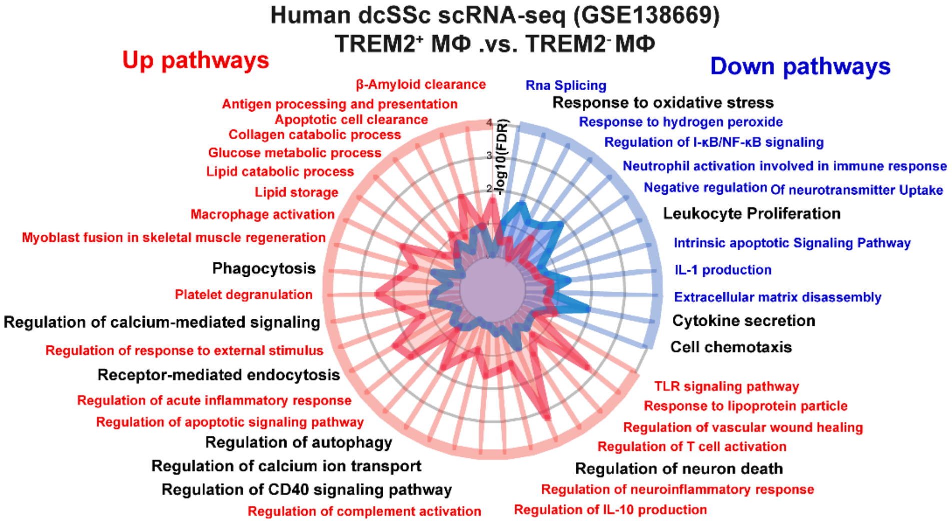

Supplementary Figure S3. The comparision of transcriptional features between TREM2$^+$ MΦs and TREM2$^-$ MΦs (Related to Figure 3).

A radar map shows pathways associated with TREM2$^+$ MΦs or TREM2$^-$ MΦs signatures in human dcSSc (GSE138669). Upregulated pathways and Down-regulated pathways in TREM2$^+$ MΦs shared with mouse skin fibrosis were highlighted in black.

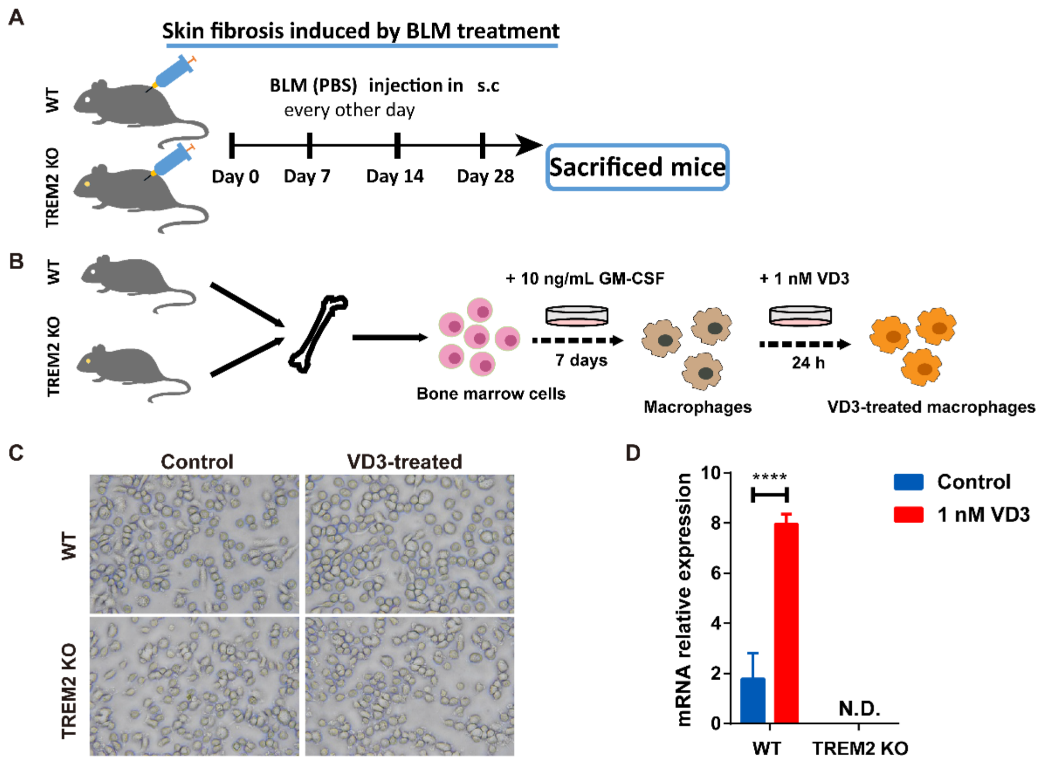

Supplementary Figure S4. VD3 induces TREM2$^+$ macrophage subpopulation (Related to Figure 5).

A. The schematic diagram for mouse skin fibrosis induced by subcutaneous BLM injection.

B. The strategy of BMDM culture *in vitro*. BM was isolated from WT or TREM2 KO mice and cultured in the presence of GM-CSF for 7 days. The cells were then stimulated with 1 nM VD3 for 24 h (DMSO stimulated as control).

C. Representative pictures (magnification 200×) of BMDM from WT and TREM2 KO mice treated or untreated by 1 nM VD3 *in vitro*.

D. RT-PCR detected the mRNA expression of *Trem2* in BMDM treated by 1 nM VD3.

Images and data shown were representative of at least 3-5 mice every group corresponding to three independent experiments. P-values were calculated with the 2-tailed Student's *t*-test, Means ± SEM are shown (ns, not significant, ****P < 0.0001).

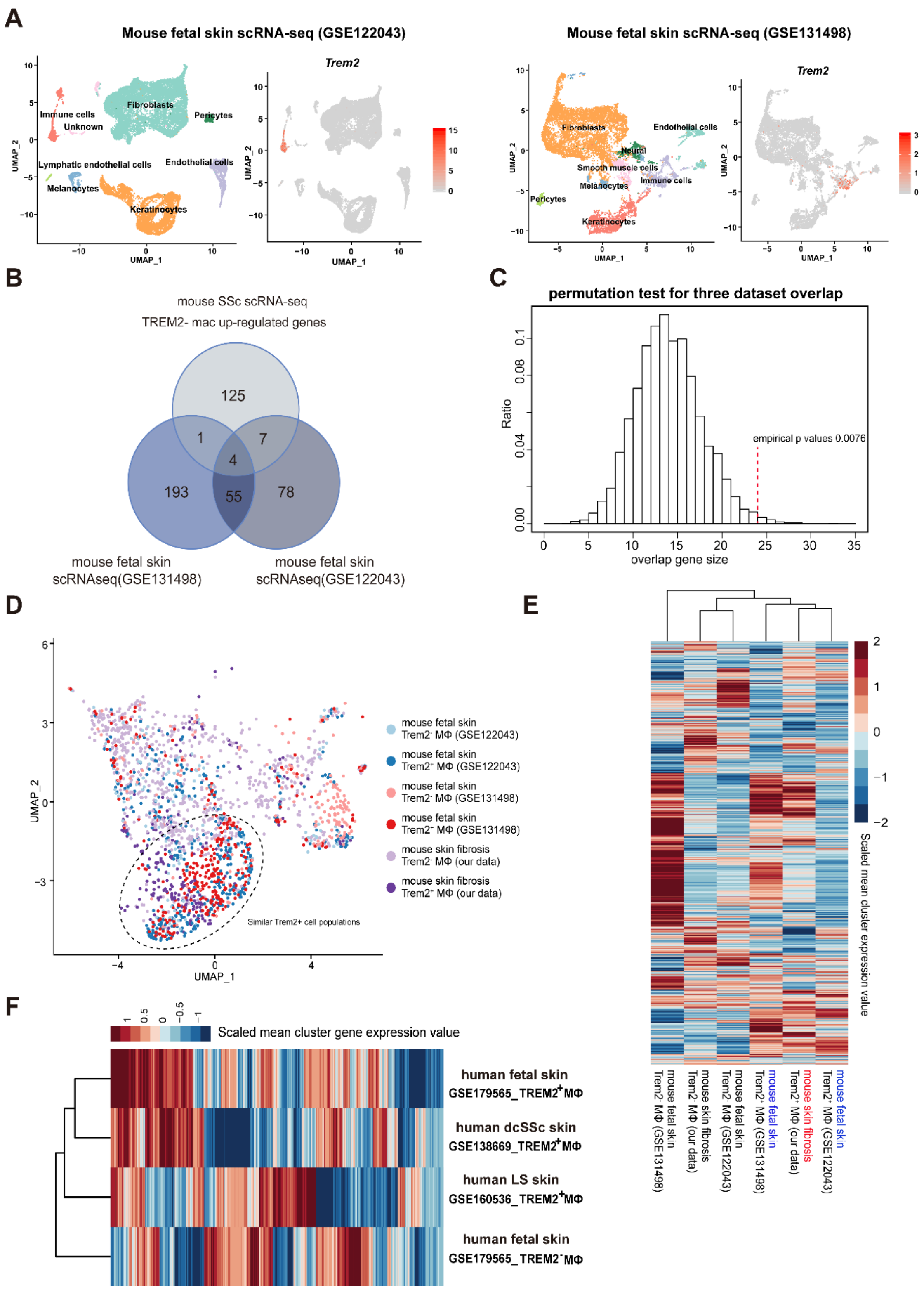

Supplementary Figure S5. TREM2$^+$ MΦs appear in fetal skin have similar program with compartments in mouse skin treated by BLM (Related to Figure 6).

A. UMAP visualization shows a analysis of mouse fetal skin from datasets (GSE122043 and GSE131498). Cells are colored by cell type, and the expression

of *Trem2* is shown in UMAP.

B. Venn diagram showing common and unique DEGs upregulated in TREM2$^-$ MΦs from mouse skin fibrosis scRNA-seq, TREM2$^+$ MΦs from mouse fetal skin scRNA-seq (GSE131498) as well as mouse fetal skin scRNA-seq (GSE122043) datasets. The 4 conserved genes were listed.

C. The analysis of random chance for overlapping among upregulated genes in TREM2$^+$ MΦs from the mouse fetal skin dataset (GSE122043 and GSE131498), as well as the mouse skin fibrosis dataset (our data). The resulting empirical p-value was 0.0076 (see methods).

D. Integration analysis for TREM2$^+$ MΦs and TREM2$^-$ MΦs from mouse fetal skin (GSE122043 and GSE131498), and mouse skin fibrosis (our data).

E. Heatmap plot showing the hierarchical clustering result of gene expression for TREM2$^+$ MΦs and TREM2$^-$ MΦs from two mouse fetal skin scRNAseq data (GSE131498, GSE122043) and mouse skin fibrosis (our scRNAseq data).

F. Heatmap plot showing the hierarchical clustering result of gene expression for TREM2$^+$ MΦs from human dcSSc scRNAseq (GSE138669), human LS scRNAseq (GSE160536), and human fetal skin scRNAseq data (GSE179565) and TREM2$^-$ MΦs from human fetal skin scRNAseq data (GSE179565).
